# Quantifying Hydrogen Peroxide in Cattle Milk via a Novel Plasmonic Nanofilm

**DOI:** 10.1021/acsomega.5c01585

**Published:** 2025-05-19

**Authors:** Upama Das, Rajib Biswas, Nirmal Mazumder

**Affiliations:** † Applied Optics and Photonics Research Laboratory, Department of Physics, 28688 Tezpur University, Tezpur , Assam 784028, India; ‡ Department of Biophysics, Manipal School of Life Sciences, 76793 Manipal Academy of Higher Education, Manipal, Karnataka 576104, India

## Abstract

Hydrogen peroxide is an illegal milk adulterant occasionally utilized as a milk preservative to extend its shelf life. While it is highly effective at reducing spoilage, its use poses significant health risks due to its high toxicity on human health, along with strong oxidative and corrosive properties. Plasmonic sensing principles offer a powerful tool for detecting hydrogen peroxide adulteration, even in trace amounts, below permissible limits. Hydrogen peroxide, being a strong oxidizing agent, binds with the functionalized nanoparticles in the sensor. This binding alters the local refractive index by reducing the number of nanoparticles, along with alteration of their shape and size. These changes can be used to quantify the amount of adulterants in milk. Taking cue from this, our work reports fabrication of a localized surface plasmon resonance (LSPR)-based milk adulterant sensor to detect hydrogen peroxide utilizing glass as the primary substrate. In this approach, silver nanoparticles were synthesized using Ocimum tenuiflorum leaves extract, and then coated with polyvinyl alcohol as a functionalizing element. When exposed to hydrogen peroxide, the immobilized nanoparticles interacted with the adulterant, causing a decrease in absorbance along with a shift in the plasmonic peak. This shift was calibrated to accurately estimate the amount of hydrogen peroxide in milk, allowing sensitive detection of the adulterant, even at trace levels much lower than the permissible limit.

## Graphical abstract

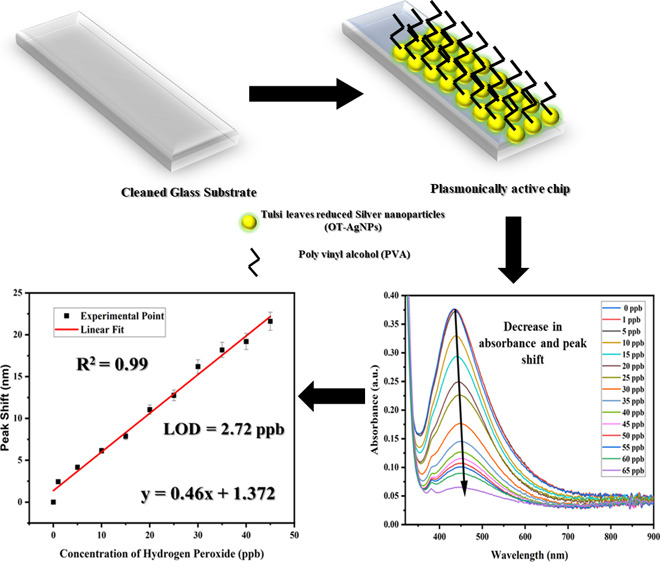

## Introduction

Hydrogen peroxide is a colorless liquid when diluted, but appears to be a pale blue in its purest form.^1^ It is an inorganic compound where two hydroxy groups (OH) are attached via a covalent oxygen–oxygen single bond. It is utilized in a diverse array of applications such as disinfectants, antiseptics, and the treatment of wastewater.^2^ Owing to its strong oxidizing properties, it can stop the microorganism’s growth in milk, thereby increasing the shelf life of various food items.^3^ However, due to its corrosive, toxic, and hazardous nature, all government food regulatory bodies have imposed strict regulations on the addition of hydrogen peroxide in food items. Its consumption can cause serious health problems such as gastrointestinal damage, oxygen embolism, respiratory distress, and cellular damage.^4,5^

Milk is a highly perishable commodity due to its rich nutritional composition, including proteins, lactose, and fats, which makes it susceptible to microbial degradation.^6^ To extend its shelf life and prevent spoilage, various chemical preservatives, including hydrogen peroxide, are often added. In the presence of hydrogen peroxide, the enzyme lactoperoxidase oxidizes the thiocyanate present in milk. The thiocyanate gets converted to hypothiocyanous acid (HOSCN), mainly in (OSCN^–^) ionic form, which reacts with free sulfhydryl groups present in milk. This reaction inhibits metabolic bacterial enzymes, and as a result, the metabolism of vital bacteria present in milk is completely blocked. They lose their ability to multiply. However, this reaction can only occur in the presence of the compound hydrogen peroxide.^7,8^ It thereby acts as an antimicrobial agent, inhibiting bacterial growth and delaying milk degradation.^9^ However, its addition poses significant health risks as excessive consumption has been linked to oxidative stress, gastrointestinal disturbances, and other adverse effects. This has led to stringent regulatory limits on hydrogen peroxide levels in milk, necessitating the development of reliable detection methods.^10^

Traditionally, various analytical techniques were utilized for the detection of milk adulterants, such as gas chromatography–mass spectrometry (GC-MS),^11^ and high-performance liquid chromatography (HPLC)^12^ offer precise detection but are often costly, labor-intensive, and require specialized expertise. Alternative methods, including the development of colorimetric assays,^13^ surface-enhanced Raman Scattering (SERS)^14^ techniques, and electrochemical methods,^15^ have been explored for hydrogen peroxide detection in milk. However, many of these approaches suffer from high detection limits, qualitative rather than quantitative results, or complex sample preparation steps for detecting trace amounts of hydrogen peroxide in complex matrices like milk. Moreover, these methods may lack the requisite cost-effectiveness, sensitivity, and selectivity to detect adulterants at trace levels.

Meanwhile, plasmonic nanostructures (NSs) offer a promising avenue for the fabrication of sensitive and rapid detection platforms.^16^ Their outstanding optical properties, characterized by localized surface plasmon resonance (LSPR), enable label-free detection with high specificity and sensitivity.^17^ By harnessing the interaction between the plasmonic NSs and target analytes, it is possible to achieve real-time monitoring with minimal sample pretreatment.^18^ Furthermore, the tunability of plasmonic nanoparticles (NPs) allows for the optimization of sensing performance, including augmented detection limit and selectivity.^19,20^

Although in the literature, there are certain methods such as colorimetric method, chromatographic analysis, fluorescent and electrochemical methods available to detect the toxic adulterants.^21,22^ Even though these methods are highly advanced and sophisticated, some of them could not even detect trace amounts of it in milk, making it necessary to fabricate a user-friendly device which could be used for on-spot and real-time assessment of the quality of milk.^21,22^ Here, the plasmonic optical sensor comes as a savior. Recently, a notable study by Semwal and Gupta fabricated a Surface Plasmon Resonance (SPR)-based sensor using fiber optic technology to detect hydrogen peroxide. The detector was composed of a gold (Au) and graphene oxide (GO) coating on the core of an unclad optical fiber, and it was covered with immobilized catalase enzyme. Au was used to enhance the stability of the sensor. While GO improved its sensitivity, catalase provided high specificity for hydrogen peroxide detection. This sensor achieved an LOD of 55 μM. However, the fabrication process of this sensor was complex, requiring multiple chemicals and intricate techniques.^23^

In light of these challenges, the fabrication of a cost-effective, easy-to-use, rapid detection method for estimating hydrogen peroxide content in milk is the need of the hour. Taking a cue from this, the reported work focuses on LSPR-based sensing techniques to enable the quantitative estimation of hydrogen peroxide concentrations in milk. Accordingly, this work introduces a novel approach for detecting hydrogen peroxide in raw milk with simplicity and efficiency, eliminating the need for a cumbersome NP preparation technique or critical milk pretreatment. The synthesis strategy is meticulously designed to intensify the NPs’ selectivity specifically for detecting hydrogen peroxide as a milk adulterant. The resulting sensing platform offers an intuitive, robust, and cost-effective means of quantifying hydrogen peroxide levels in milk.

## Materials and Methods

### Chemicals

Fresh cow milk is procured from a nearby cattle farm. Ocimum tenuiflorum (OT) (Tulsi) leaves were obtained from the Tezpur University Campus, Tezpur, Assam, India. Silver Nitrate (AgNO_3_) was purchased from Thermo Fisher Scientific. (3-Aminopropyl)-triethoxysilane (≥98.0%) (APTES), polyvinyl alcohol (PVA), Bovine Serum Albumin (BSA), sodium borohydride (NaBH_4_), sodium hydroxide (NaOH), dextrose, salicylic acid, and ammonium phosphate were obtained from Merck, USA. Trichloroacetic acid was from Qualigens. Melamine (extra pure) and urea (extra pure) were obtained from Loba Chemie. Formaldehyde (37%) and hydrogen peroxide (30%) were obtained from Emplura. Here, melamine, urea, formaldehyde, dextrose, salicylic acid, and ammonium phosphate were used for the selectivity study. The microscopic glass substrate purchased from a local surgical store was utilized for the fabrication of the chip.

Acetone, methanol, and ethanol were obtained from Qualigens and were utilized for cleaning purposes. All glassware and magnetic stirrers used in synthesis were dipped in aqua regia and chromic acid overnight and rinsed in distilled water and preserved until dry. Distilled water (DW) was used for the synthesis.

### Instrumentation

UV–visible spectrophotometer: Thermo Scientific GENESYS 180; transmission electron microscope (TEM): Tecnai G2 20 S-TWIN; field emission scanning electron microscope (FESEM): GEMINI 500; X-ray powder diffractometer (XRD): BRUKER D8 ADVANCE ECO; Atomic Force Microscopy (AFM): NTEGRA Vita device from NTMDT & Asylum Research MFP-3D-BIO.

Centrifuge machine: Eppendorf 5430R; weighing machine: METTLER TOLEDO ME204; oven: EQUITRON; tabletop pH meter: EUTECH pH 700; and magnetic stirrer: SPINOT-TARSONS.

### Synthesis of PVA-Functionalized AgNPs

Ten mM AgNO_3_ solution was prepared by adding 0.169 g to 100 mL of DW, which was stirred at room temperature for a duration of 10 min at a rate of 600 rpm. Following this, the tulsi leaves extract was prepared. Accordingly, 5 g of tulsi leaves was chopped into small pieces, washed properly, and kept in an oven to dry at 80 °C for 2 h. These dried tulsi leaves were crushed and added to 100 mL of DW. The solution was then heated at 100 °C for 2 h. Using a Whatman filter paper 1, this extract was filtered twice.^24^ For functionalization of NPs, a 1% PVA solution was prepared, for which 1 g of PVA was added to 100 mL of DW and dissolved by stirring it at 60 °C for a duration of 3 h at a rate of 900 rpm.

At first, 40 mL of 10 mM AgNO_3_ solution was stirred continuously, to which 5 mL of tulsi leaves extract was added dropwise, and the resulting solution was stirred at 900 rpm at 80 °C for a duration of 30 min. The color of the solution slowly changed to yellow, indicating the formation of NPs in the solution. The pH of the solution was then adjusted to 7 by adding 0.1 M NaOH solution under continuous stirring at 900 rpm. The yellow color of the resulting solution indicated the formation of AgNPs.^24,25^ Following this, the functionalisation of synthesised AgNPs with PVA was performed directly on the glass substrate.

### Fabrication of the Sensor

To fabricate the plasmonic chip, a microscopic glass slide was chosen as the substrate to enable uniform layer-by-layer deposition of functionalized tulsi leaves reduced in silver nanoparticles (OT-AgNPs) on the glass substrate. Using a glass cutter, the slides were cut into dimensions of 0.8 × 5 cm. These glass chips were then submerged in a methanol/water mixture (1:2 ratio) to remove surface impurities. Following this, the substrates were sonicated for 20 min and rinsed thoroughly with DW four times to eliminate any residual methanol. The slides were further sonicated in DW for an additional 15 min to ensure complete cleanliness.^26,27^

Next, the cleaned glass substrates were immersed in a 1% solution of APTES at 60 °C for 1 h to enhance adhesion of the functionalized AgNPs. Any unbound APTES was removed by repeated washing with DW. The glass substrates were then dipped in OT-AgNPs for 24 h at room temperature, allowing the NPs to self-assemble into a uniform layer. Following this, the NP-coated substrates were immersed in a 1% PVA solution for 1 h for proper functionalisation of NPs and in order to improve their stability.^26,27^

After 25 h, a pale-yellow tint appeared on the glass slides, indicating successful encapsulation of the NPs on the glass substrate. The slides were then washed to remove any unbound particles and subsequently immersed in a 1% BSA solution for 30 min to effectively encapsulate the LSPR chip. This process ensured a stable and sensitive detection platform for hydrogen peroxide in milk.^26,27^ The steps are schematized in Figure 1.

**Figure 1:**
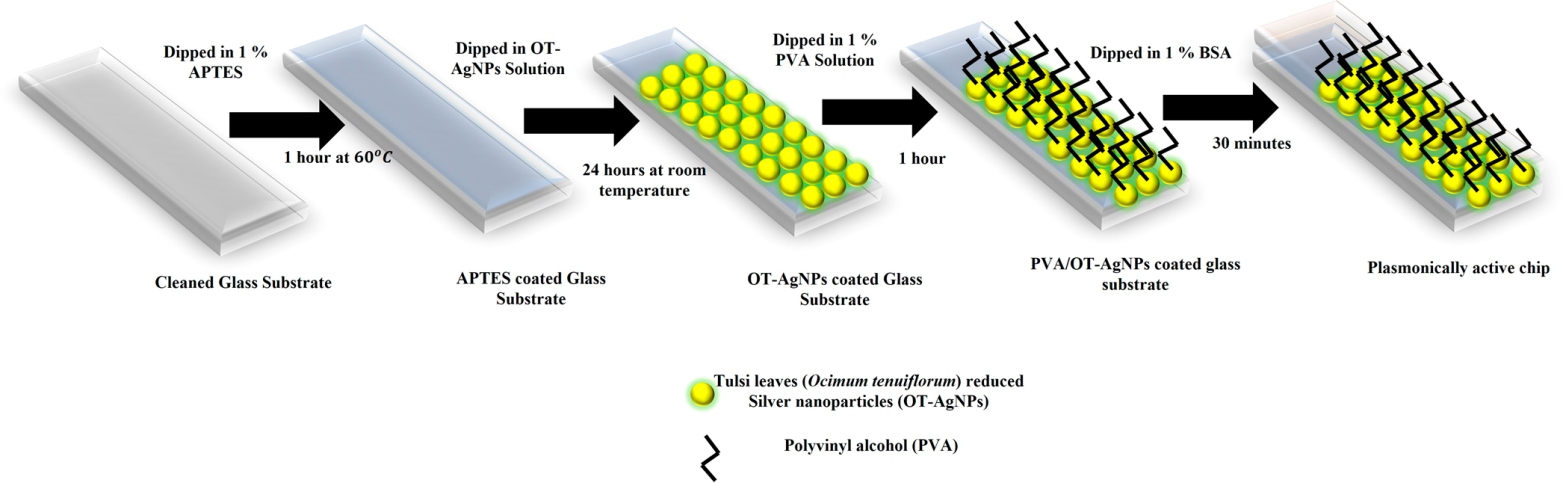
Schematic representation of fabrication of the PVA-AgNPs LSPR sensor.

### Pretreatment of Milk

Milk is a complex matrix containing high levels of fats and proteins, which can interfere with detection methods and lead to false positives or negatives. To eliminate these potential inaccuracies, the solid components of milk are removed, and only the supernatant is used for the detection process. Before pretreatment, milk samples were spiked with varying concentrations of hydrogen peroxide.^13^

For the pretreatment, 200 mL of milk was placed in a beaker and heated to 60 °C. Once the desired temperature was reached, 50 mL of a 10% trichloroacetic acid solution was added while continuously stirring until curdling occurred. The curdled mixture was then transferred to centrifuge tubes and centrifuged twice at 7000 rpm for 30 min, resulting in the precipitation of solid components. The clear supernatant was carefully separated for further analysis.^13^

To attain a neutral pH, 3 M NaOH was added to the supernatant. The solution was then filtered twice—first using Whatman filter paper No. 1 and a 0.22 μm filter—to remove any remaining precipitates. The purified supernatant was used for the detection process.^13^

## Results and Discussion

### Characterization of Synthesized Silver Nanoparticles

To determine the plasmonic characteristics of the synthesized NPs, UV–visible analysis was performed, where the NPs displayed an intense, broad absorption peak at 434 nm. The obtained broad peak indicated the polydisperse nature of the synthesized NPs (Figure 2a).^28^

**Figure 2:**
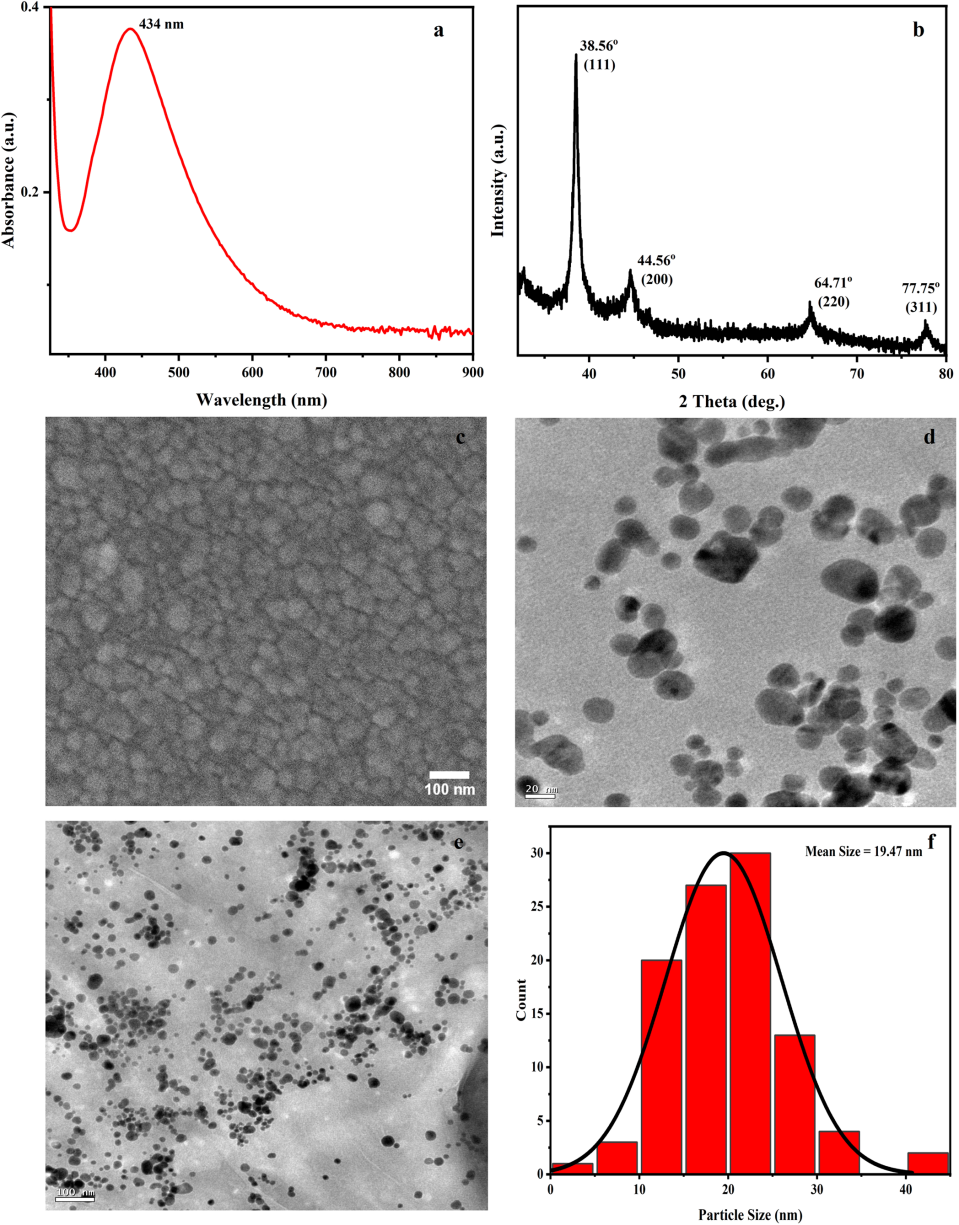

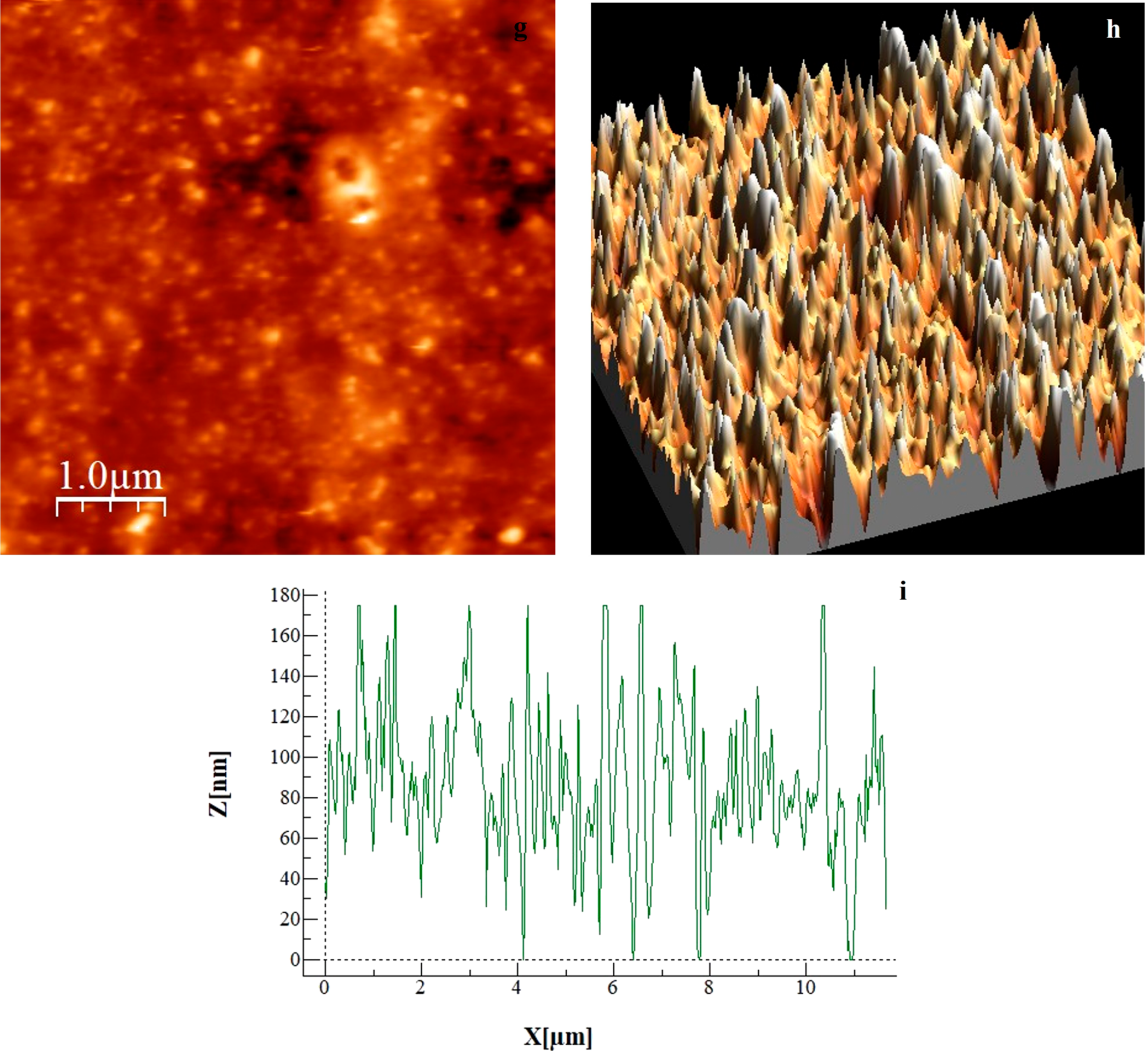
Characterization of PVA-OT/AgNPs (a) UV–vis spectrum, (b) XRD diffractogram, (c) FESEM image, (d) TEM image (under high magnification), (e) TEM image (under low magnification), (f) size distribution profile from TEM study, (g) AFM image (2D representation), (h) AFM image (3D representation), and (i) height distribution profile (thickness) of PVA-AgNPs across the LSPR chip from AFM study.

To determine the structural characteristics of synthesized NPs, XRD characterization was performed. Four distinct diffraction peaks were formed at positions 38.56, 44.56, 64.71, and 77.75°, corresponding to the diffraction planes (111), (200), (220), and (311) planes of Ag, which confirms the face-centered cubic (FCC) crystalline nature of synthesized AgNPs (Figure 2b).^29^

FESEM and TEM characterization was done to scrutinize the morphological characteristics of the synthesized NPs. FESEM images revealed the average diameter of the NPs was found to be approximately 30 nm, where each particle ranges between 10 and 40 nm (Figure 2c).^28,30^ Likewise, TEM analysis confirmed that the synthesized NPs were of size approximately, 19.47 nm, with particle sizes spanning between 3 and 45 nm. Moreover, the morphological examination depicted a predominantly spherical configuration of the synthesized NPs (Figure 2d–f).^30^

AFM analysis unveiled a homogeneous distribution of a thick layer of NPs across the surface of the glass chip. Furthermore, a profile study was performed to analyze the thickness of the NP distribution, revealing a range between 5 and 170 nm. This consistency suggests the presence of a thick, triple-layered NP along with PVA capping on the chip surface, underscoring the precision and uniformity achieved in the NP deposition process for the subsequent application (Figure 2g–i).^31–52^

### LSPR-Based Plasmonic Sensing

#### Mechanism

Tulsi leaves are a rich source of bioactive antioxidants, including polyphenols, flavonoids, tannins, and terpenoids, which play a crucial role in the synthesis of AgNPs.^33^ These biomolecules act as natural reducing agents, facilitating the reduction of silver ions (Ag^+^) into zerovalent Ag atoms. Along with reduction, the phytochemicals present in Tulsi extract also function as stabilizing and capping agents, preventing NP aggregation and ensuring their uniform dispersion. This dual role of Tulsi-derived compounds not only simplifies the synthesis process but also imparts biocompatibility to the NPs, making them suitable for sensor applications.^33^ Particularly, a flavonoid present in tulsi leaves known as Quercetin plays a significant role in the synthesis of AgNPs due to its strong reducing, stabilizing, and capping properties. Structurally, quercetin contains carbonyl (−C=O) and multiple hydroxyl (−OH) functional groups, which make it an effective electron donor. During AgNPs synthesis, quercetin interacts with Ag^+^ ions, reducing them to metallic silver (Ag^0^) through electron transfer. This reduction process is facilitated by the antioxidant nature of quercetin, which neutralizes silver ions and promotes their nucleation and growth into NPs.^34,35^

This compound also acts as a strong capping and stabilizing agent, preventing NP aggregation. The hydroxyl groups present in quercetin form strong interactions with the surface of newly synthesized AgNPs, creating a protective organic layer. This coating ensures uniform nanoparticle dispersion, enhances colloidal stability, and controls particle size and shape.^36^

Once synthesized, AgNPs are immobilized onto a glass substrate to create a stable and reproducible plasmonic sensing platform. To enhance their sensitivity and specificity, the NPs are coated with a PVA layer. This plasmonically active chip was then placed inside a quartz cuvette containing hydrogen peroxide spiked milk samples. Here, PVA serves as a functional interface, offering abundant hydroxyl groups that facilitate the binding of hydrogen peroxide molecules. This interaction ensures that hydrogen peroxide comes into direct contact with the NP surface, allowing efficient sensing.^37^

Hydrogen peroxide, being a potent oxidizing agent, interacts with the surface-bound AgNPs at these PVA-active sites, inducing oxidative transformations. This interaction alters the physicochemical properties of the NPs, including their size, shape, and surface charge.^38^

When PVA-capped AgNPs are immobilized on a substrate, the LSPR peak shift in response to the presence of hydrogen peroxide occurs due to changes in electron density, NP oxidation, and local refractive index variations. Upon exposure to hydrogen peroxide, the oxidation of AgNPs reduces the free electron density. However, at higher concentrations of hydrogen peroxide, the surface modifications and oxidation byproducts alter the local refractive index, leading to a redshift. Unlike colloidal AgNPs, where aggregation plays a key role, immobilized NPs primarily respond through plasmonic changes at the interface, influenced by the substrate material and interaction strength.^39–43^

By monitoring these plasmonic shifts, a precise and quantifiable correlation between the hydrogen peroxide concentration and optical response can be established. This LSPR-based detection mechanism provides a rapid, label-free, and highly sensitive approach for real-time monitoring of hydrogen peroxide, ensuring accurate assessment even at trace levels^39–43^ (Figure 3).

**Figure 3:**
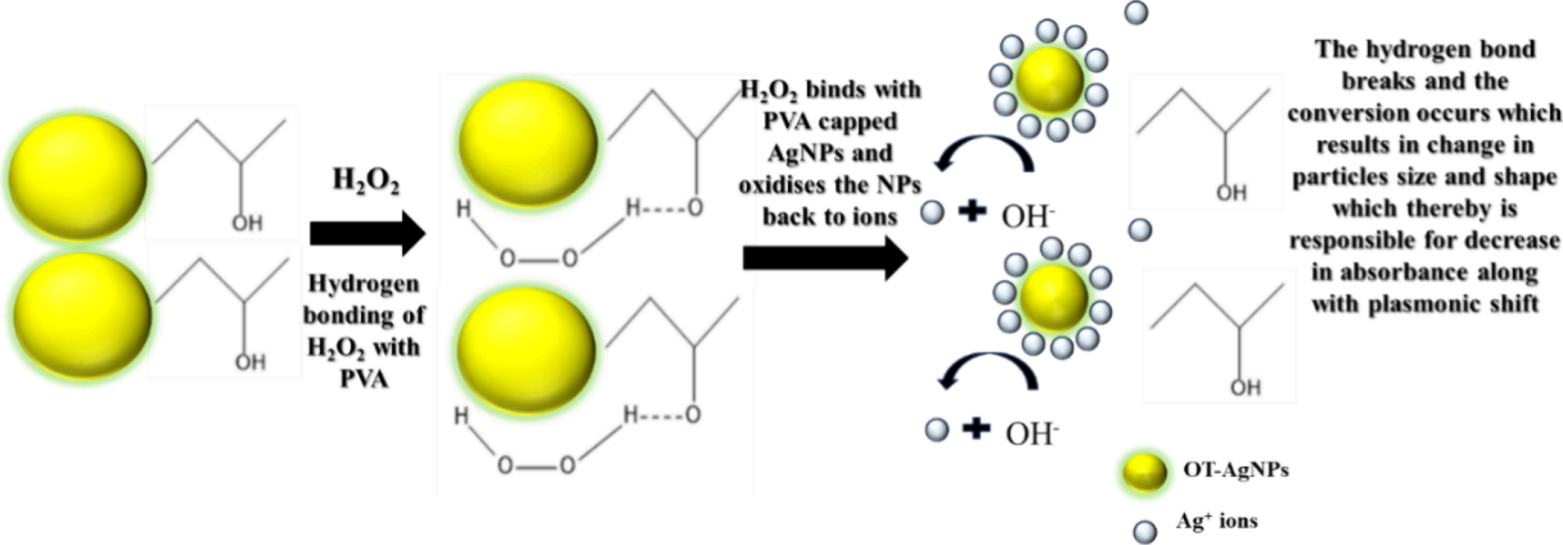
Pictorial representation of the detection mechanism for sensing of hydrogen peroxide in milk.

### Selectivity Study

A selective analysis was performed employing UV–vis analysis to evaluate the interference of common milk adulterants in the sensing process. The study was performed with the commonly used adulterants such as urea, melamine, dextrose, ammonium phosphate, salicylic acid and formalin. Milk samples were spiked with a 225 ppb concentration of each adulterant and a 45 ppb concentration of hydrogen peroxide to perform the selectivity test. Intriguingly, the analysis revealed that only hydrogen peroxide induced a significant shift in the absorbance peak, while other adulterants exhibited a very minimal influence. This is because other adulterants do not interfere with the encapsulated NPs and thus do not cause any significant shift in the LSPR band of the AgNPs. Notably, hydrogen peroxide demonstrated the most pronounced response in terms of the peak shift compared to its counterparts present in milk. This significant observation underscores the efficacy of the functionalized NPs in facilitating the precise and selective detection of hydrogen peroxide within milk samples (Figure 4).

**Figure 4:**
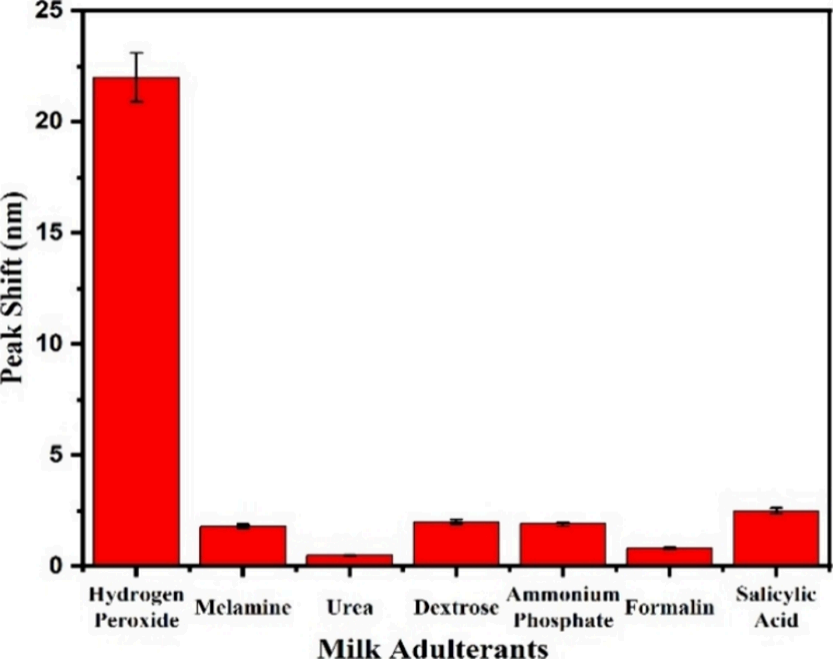
Histogram plot representing the selectivity test of the plasmonic chip in the presence of other interfering adulterants.

### Sensor Performance Metrics

Figure 5a displays UV–vis absorbance spectra, which show a substantial decrease in absorbance along with a shift in the plasmonic peak corresponding to an increment in the concentration of hydrogen peroxide. This is caused by the interaction between the surface-functionalized immobilized NPs with the adulterant hydrogen peroxide. This results in a change in the local refractive index of the media caused by the decreasing number of particles and also by the change in shape and size of NPs upon interaction with the hydrogen peroxide present in the milk. As shown in Figure 5b,c, a linear correlation was established between the observed decrease in absorbance/peak shift with an increase in the amount of hydrogen peroxide, respectively. The graph in Figure 5c enabled the determination of the LOD, which has been calculated from eq 1 by utilizing the slope (*S*) of the calibrated graph and standard error (σ).^13^

LOD=3σ/S 1

**Figure 5:**
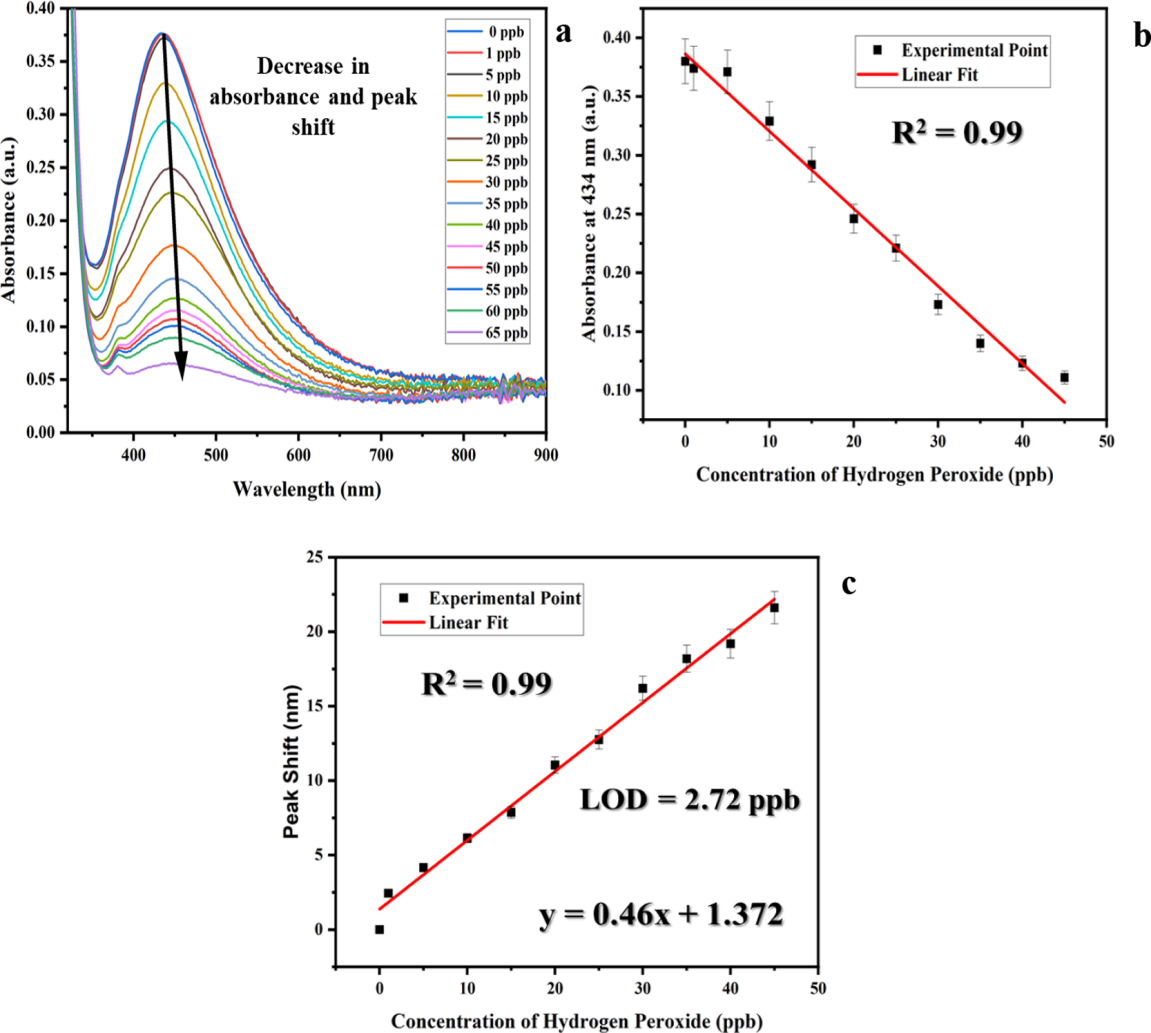
(a) UV–vis plots depicting peak shift and decrease in absorbance with changing concentrations of hydrogen peroxide in milk, calibration plot between (b) absorbance vs concentration of hydrogen peroxide, and (c) peak shift vs concentration of hydrogen peroxide.

By utilizing this equation, the LOD has been found to be 2.72 ppb.^44^ Notably, this value highlights the exceptional sensitivity of the developed sensing platform, exceeding the stringent limits established by regulatory agencies. The sensing probe demonstrated a dynamic detection range of 1 to 45 ppb, with a sensitivity of 0.46 nm/ppb change in concentration of hydrogen peroxide, reflecting its capability to detect even trace amounts of hydrogen peroxide concentration with remarkable precision (Figure 5).

Furthermore, the recovery efficiency of the fabricated device was assessed, and it emerged as 97–108%. This highlights the sensor’s reliability and reproducibility in detecting hydrogen peroxide in milk supernatant with higher accuracy.^45^ Table 1 highlights the recovery and LOD of the reported sensor compared to the previously reported studies.

**Table 1:** Comparative Study of Hydrogen Peroxide Sensing Using Plasmonic Nanoparticles

sensing element	recovery	limit of detection/lowest limit of sensing	references
cellulose nanowhiskers-AgNPs	98%	0.476 ppb	^46^
sericin-AgNPs	104–117%	1.5 ppm	^47^
Sargassum boveanum-AgCl NPs	98.58–100.80%	0.29 ppb	^48^
Benincasa hisipida-AgNPs	not performed	34.014 ppm	^49^
cotton leaves-AgNPs	92%	8.46 ppm	^13^
AuPt NP-modified electrode	82–116%	0.085 ppm	^15^
bimetallic Pd–Ag NP-functionalized r-GO	96.76–102.42%	23.81 ppb	^50^
AgNP-modified r-GO	97.20–99.41%	24.83 ppb	^51^
Au-and graphene oxide coated on an unclad optical fiber core	not performed	1.87 ppm	^23^
PVA-OT-AgNPs	**97–108%**	**2.72 ppb**	**reported work**

Along with a qualitative study, this experimental setup can also be utilized for quantification in order to determine the amount of hydrogen peroxide in milk. To validate this, various numbers of experiments were performed in spiked samples, and the error associated with the calibrated graphs was obtained. The error ranged from 1.33 to 8%, indicating that the fabricated sensor system can provide accurate quantitative assessments of the hydrogen peroxide concentration in milk. The process entails measuring the peak shift value, which is then applied to the linear calibration equation to accurately determine the hydrogen peroxide concentration with minimal error. This enables precise quantitative analysis of hydrogen peroxide, even at trace levels, in milk samples (Table 2).

**Table 2:** Quantitative Estimation of Hydrogen Peroxide in Milk

sl. no.	peak shift (*y*) (nm)	theoretical value of concentration (*x*_0_) (ppb)	experimental value of concentration (*x*) (ppb)	error %^a^
1	2.61	2.5	2.7	8.00
2	4.78	7.5	7.4	1.33
3	6.98	12.5	12.2	2.40
4	9.61	17.5	17.9	2.28
5	12.04	22.5	23.2	3.11

a $\mathrm{error \%}=\left|\frac{x_{0}-x}{x_{0}}\right|\times 100$ .

## Conclusions

This study reports the fabrication of a highly selective and sensitive plasmonic sensor for the detection of hydrogen peroxide in milk, representing a significant advancement in milk safety monitoring. Utilizing comprehensive characterization techniques, such as UV–vis spectroscopy, XRD, TEM, FESEM, and AFM, the plasmonic, structural orientation, and morphological aspects of the synthesized NPs were elucidated. NPs functionalized with PVA exhibited a strong affinity for the target analyte, hydrogen peroxide. This led to distinct plasmonic peak shifts, as confirmed by UV–vis spectroscopy. The sensor demonstrated exceptional performance with an LOD of 2.72 ppb for hydrogen peroxide, surpassing the stringent thresholds set by regulatory agencies. Recovery efficiency of 97–108% shows the reliability of the system for repeated and quantitative measurements. Additionally, the innovative design, leveraging the LSPR technology, offers high reproducibility and real-world applicability. Overall, this LSPR-based sensor offers a user-friendly, eco-friendly, and highly accurate solution for on-site milk quality monitoring. Its robust performance and simplicity in fabrication position it as a promising tool for combating milk adulteration.

## Data Availability

All the data set related to the research work performed has already been added to the manuscript.
